# Deciphering the Impact of a Bacterial Infection on Meiotic Recombination in *Arabidopsis* with Fluorescence Tagged Lines

**DOI:** 10.3390/genes11070832

**Published:** 2020-07-21

**Authors:** Ariane Gratias, Valérie Geffroy

**Affiliations:** 1 Institute of Plant Sciences Paris-Saclay (IPS2), Université Paris-Saclay, CNRS, INRAE, Univ Evry, 91405 Orsay, France; ariane.gratias-weill@universite-paris-saclay.fr; 2 Institute of Plant Sciences Paris-Saclay (IPS2), Université de Paris, CNRS, INRAE, 91405 Orsay, France

**Keywords:** biotic stress, meiotic recombination, NLR genes, *Arabidopsis*, Fluorescent Tagged Lines, *Pseudomonas syringae*

## Abstract

Plants are under strong evolutionary pressure to maintain surveillance against pathogens. One major disease resistance mechanism is based on NB-LRR (NLR) proteins that specifically recognize pathogen effectors. The cluster organization of the NLR gene family could favor sequence exchange between NLR genes via recombination, favoring their evolutionary dynamics. Increasing data, based on progeny analysis, suggest the existence of a link between the perception of biotic stress and the production of genetic diversity in the offspring. This could be driven by an increased rate of meiotic recombination in infected plants, but this has never been strictly demonstrated. In order to test if pathogen infection can increase DNA recombination in pollen meiotic cells, we infected *Arabidopsis* Fluorescent Tagged Lines (FTL) with the virulent bacteria *Pseudomonas syringae*. We measured the meiotic recombination rate in two regions of chromosome 5, containing or not an NLR gene cluster. In all tested intervals, no significant difference in genetic recombination frequency between infected and control plants was observed. Although it has been reported that pathogen exposure can sometimes increase the frequency of recombinant progeny in plants, our findings suggest that meiotic recombination rate in *Arabidopsis* may be resilient to at least some pathogen attack. Alternative mechanisms are discussed.

## 1. Introduction

Pathogens cause important yield losses to agriculture and are also a key driver of biological diversity in natural plant ecosystems. In this coevolution arm race, plants are under strong evolutionary pressure to maintain efficient surveillance against pathogens. The plant immune system consists of two major branches acting as a multi-layered defense system: (i) the Pathogen-Associated Molecular Pattern (PAMP) Triggered-Immunity (PTI) and (ii) the Effector-Triggered Immunity (ETI). The PTI signaling recognizes conserved PAMPs shared by many microbes, through the action of Pattern-Recognition Receptors (PRR) located at the plasma membrane. To suppress PTI, successful pathogens have evolved virulence factors that act in host cells. In turn, plants have evolved intracellular immune receptors, the so-called disease resistance (*R*) genes, that specifically recognize pathogen effectors and activate the ETI signaling [1,2].

*R*-gene-dependent recognition of pathogen effectors is a cornerstone of plant defense against pathogens. The largest class of *R* genes encode intracellular nucleotide-binding/leucine-rich repeat (NLR) receptors. Plant NLRs can be grouped into two major families based on their N-terminal domains: the CC-NLR (CNL) and the TIR -NLR (TNL), that are broadly defined by their N-terminal coil-coiled (CC) domains or Toll/interleukin-1 receptor (TIR). NLR can detect directly or indirectly the polymorphic, strain-specific pathogen effectors and transfer the signal to the downstream defense genes, triggering hypersensitive cell death [3,4,5].

Genome sequencing revealed that NLR-encoding genes have highly expanded in higher plant genomes and constitute one of the largest and most diversified gene families often with hundreds of members [3]. Most NLR genes are organized in clusters that comprise either several copies of homologous NLR genes arising from a single gene family (homogenous cluster) or distantly phylogenetically related NLR sequences (heterogeneous clusters) [6,7]. They may also contain unrelated genes interspersed between the NLR genes. The coexistence of these two types of *R* clusters suggests that different mechanisms are responsible for NLR gene evolution such as tandem duplication of a paralogous sequence via unequal crossovers (COs) but also ectopic recombination in which single genes or small groups are transposed to distant locations. Nonhomologous end joining (NHEJ), another DNA repair process that can ligate DSBs in the genome without a homologous template, has been proposed to be involved in this ectopic recombination process [8,9,10]. Concerning a single NLR cluster, genic and intergenic sequence repeats within NLR clusters provide a structural environment that permits mispairing of nonallelic but related sequences during meiotic recombination, giving rise to unequal COs. These unequal COs contribute to the evolution of NLR clusters not only by duplicating genes and leading to local tandem duplications of related NLR within a cluster but also by deleting genes or by fusing genes which can lead in some cases to new R specificities [11,12,13,14]. Consequently, *R* gene evolution is thought to be facilitated by their cluster organization which permits sequence exchange via recombination mispairing ([3,15] and for review see [7]) and thus, by generating the diversity on which natural selection can act, recombination plays a central role in the evolution of plant immunity.

In different plant species, classical genetic analysis based on noninfected segregating populations such as F2 or RILs have shown that NLR clusters can be either coldspot or hotspot of recombination [4,16,17]. This probably reflects the variety of chromosome locations of *R* gene clusters that can be either pericentromeric or subtelomeric. In addition, the low rate of recombination could also be a consequence of haplotype and structural hybridity at *R* gene clusters [17]. Similarly, SNP mapping of meiotic crossovers in two major NLR clusters located on *Arabidopsis* chromosomes 1 and 5 revealed heterogeneity in local crossover frequency. Indeed, a subset of NLR genes overlap with strong crossover hotspots while other NLRs correspond to coldspots [18]. Since recombination increases genetic diversity, recombination hotspots in (immunity genes such as) NLR genes could generate variations that can be beneficial to plants [18].

Strikingly, Homologous Recombination (HR), a DNA repair process, is activated in somatic cells of pathogen-infected plants, suggesting that HR is a common feature associated with biotic stresses. Indeed, using recombination substrates harboring two truncated but overlapping parts of a reporter gene (GUS or LUC), an increase of somatic HR frequency has been reported not only in *Arabidopsis* plants treated with flg22, a bacterial PAMP, or infected with the oomycete *Hyaloperonospora arabidopsidis* but also in tobacco plants infected with either TMV (Tobacco Mosaic Virus) or ORMV (Oilseed Rape Mosaic virus) [19,20,21]. Consistently, biotic stresses are also associated with an increase of Double-Strand Breaks (DSBs), the substrate of DNA repair processes such as HR [22]. For example, Song et al. reported that a variety of plant pathogens with diverse lifestyles, including bacteria, oomycete and fungus, induce DSBs in host plant cells during compatible interactions [23]. Other evidence of the link between biotic stresses and DSBs comes from mutants constitutively expressing defense genes and presenting an increase in spontaneous DSBs. This is particularly well exemplified by the accumulation of DNA damages detected in the leaves of *sni1* (suppressor of *npr1*; a master regulator of the Salicylic Acid (SA)-mediated defense responses), in which the Pathogenesis-related protein, PR1, is overexpressed [24]. In somatic cells, since accidental DSBs constitute the most cytotoxic damages, DSBs monitoring is critical to orchestrating DNA repair pathways in order to sustain genome stability. This is particularly true in somatic cells of infected plants where increases in HR reflect the increase of spontaneous DSBs. Moreover, the increase of DSBs in infected plants was also shown to be associated with the upregulation of several key DNA repair genes. Indeed, local exposure to ORMV in *Arabidopsis* led not only to an increase in DSBs and but also to an upregulation of the DNA repair protein RAD51 (RADIATION SENSITIVE 51) which is a key player in HR [25]. Altogether these results indicate that both DSBs and HR required to repair these deleterious DNA damages are induced in somatic cells during the biotic stresses.

After pathogen infection, a Systemic Recombination Signal (SRS) has been evidenced, that is generated locally at the site of infection and is able to propagate to noninfected tissues, leading to a systemic increase in the somatic recombination frequency. Indeed, by either using grafting experiments or by removing the infected leaves a few hours after virus inoculation, Kovalchuk’s group showed that *Arabidopsis* and Tobacco plants exposed to compatible viral infection (ORMV and TMV, respectively) present an increase of somatic HR frequency, not only in infected tissues but also in noninfected tissues, suggesting the existence of an SRS [21,25]. And strikingly, this SRS, elicited by the virus at the infection site and the nature of which is as yet unknown, can spread into distant tissues faster than the virus [21].

Since reproductive tissues appear late in the plant lifecycle, it is highly conceivable that the SRS could reach the floral organs, leading to an increase of the DNA recombination in meiocytes. In meiotic cells, the DNA recombination is initiated by programmed DSBs, actively produced by Spo11-topoisomerase-like complexes. These DSBs are subsequently repaired by certain mitotic proteins of the Homologous Recombination (HR) machinery and specific meiotic actors, such as DMC1. This drives tightly controlled repair using a chromatid from the homologous chromosome and generates crossovers [26]. Interestingly, scarce data supporting this idea showed that a higher frequency of plants in the progeny of infected plants presents genomic rearrangements compared to the progeny of noninfected plants. Exposure to the SRS signal in TMV-infected tobacco carrying a LUC reporter gene caused a three-fold increase in the number of plants recombined in the LUC transgene in the progeny of infected plants [21]. Similarly, RFLP analysis performed in tobacco to detect changes in the homologs of resistance genes carrying the homology of the LRR region of the *N*-gene resistance gene, responsible for resistance to TMV, provide evidence for stress-induced rearrangement of *R* clusters. Indeed, TMV infection of susceptible plants resulted in an approximately six- to eight-fold increase in restructuring events detected in homologs of the LRR region of *N*-gene in the progeny of infected, versus noninfected plants, whereas increase instability was not detected at other loci [27]. Consistently, with these results suggesting that this could be a consequence of increased meiotic recombination due to the biotic stress and the resulting SRS, a significant increase of meiotic abnormalities such as the formation of chromosomal bridges, elimination and/or lagging chromosomes were described in virus-infected tomato and barley cultivars [28].

Meiotic COs by reshuffling parental genetic information constitutes an important mechanism for generating alleles with new functions. Since the SRS can propagate in the plant, triggering an increase of somatic HR, and can potentially reach the floral organs, it could constitute an adaptive mechanism to generate new *R* specificities in plants, by increasing the meiotic recombination. Demonstrating that pathogen infection could lead to an increase of meiotic recombination is of major importance at the evolutionary level, since it would reinforce the possible link between the perception of stress and the generation of diversity. However, to date, there is no direct evidence that pathogen infection in vegetative organs can impact the meiotic process by increasing COs in flowers. In this paper, we address this question by analyzing the consequence of pathogen infection on the genome and more specifically on NLR clusters, in germline cells during meiosis. To that end, we took advantage of *Arabidopsis* lines called Fluorescent Tagged Lines (FTL) [29,30], since these transgenic lines constitute a powerful tool to study thousands of individual meiotic events directly on pollen tetrads, without analyzing the genome of the progeny. Here, the effect of *Pseudomonas syringae* on meiotic recombination frequency in regions with or without NLR clusters was estimated in the pollen of two *Arabidopsis* FTL lines carrying appropriate fluorescent markers. After infection of *Arabidopsis* leaves with the virulent bacteria, we found no significant increase in the rate of meiotic recombination, whatever the interval. We comment on the biological implications of these findings and discuss alternative explanations.

## 2. Materials and Methods

### 2.1. Identification of Arabidopsis NLR Genes

We combined published annotations of the *Arabidopsis* NLR family [31] with our manual searches and selected two intervals located on chromosome 5, from 2.3 to 5.4 Mb (3.1 Mb) and from 18.1 to 25.7 Mb (7.6 Mb) and containing, respectively, 1 and 21 NLR genes, and for which FTL lines were already available (see next section).

### 2.2. Arabidopsis Fluorescent Tagged Lines

**Figure 1:**
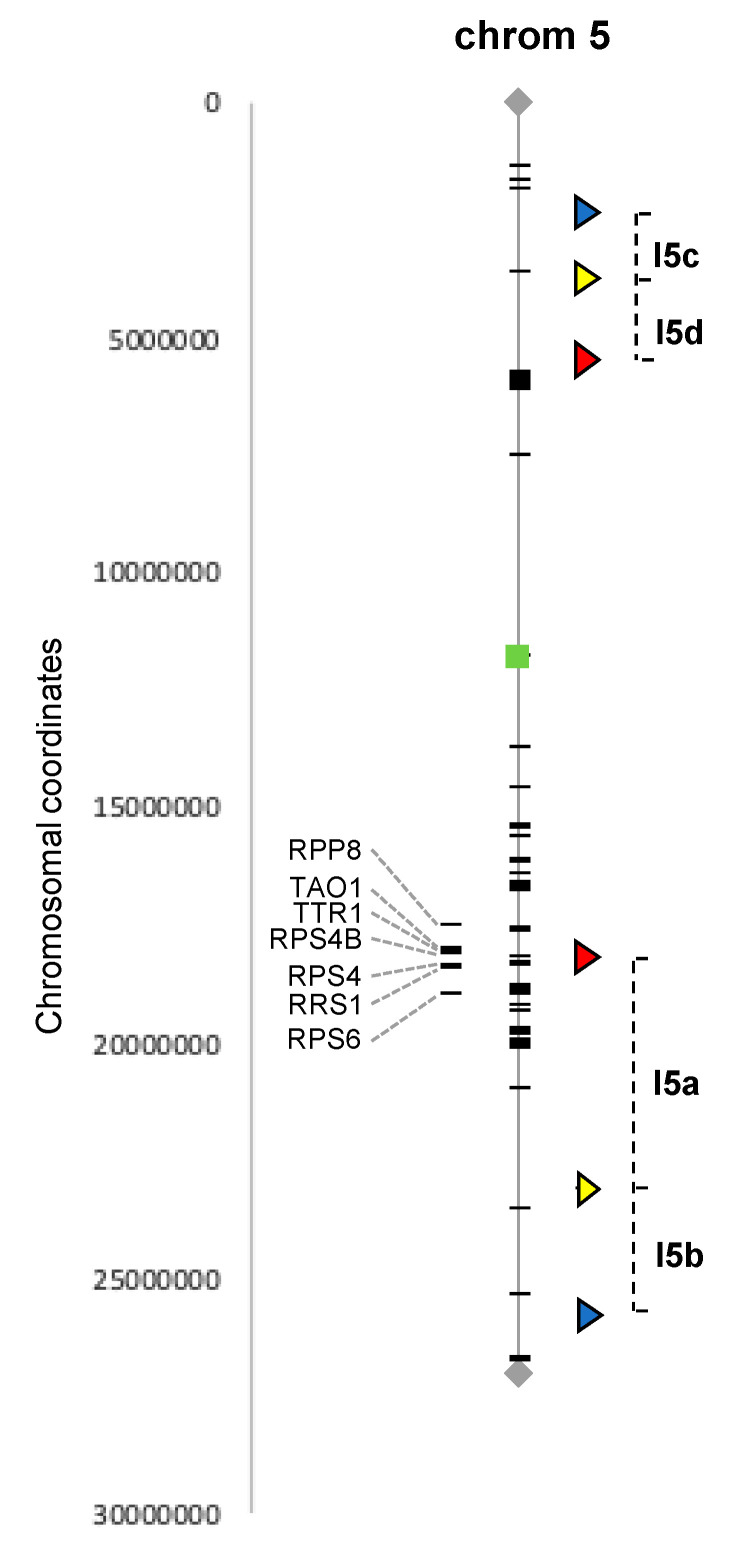
Distribution of NLR genes and Fluorescent Tags on the *Arabidopsis* chromosome 5. The chromosome with its centromere (green square) is delimited by the telomeres (black lozenge) at each end. The NLR encoded-genes (black dashes) are localized on the gray thin line of the chromosome or on the left side for NLR *R* genes with known biological functions. The Fluorescent Tags (triangle) and the intervals determining the Fluorescent Tagged Lines (FTL) I5ab and I5cd are indicated on the right side of the chromosome.

To gain insight into DNA meiotic recombination in flowers of infected plants, Fluorescent Tagged Lines developed by Gregory P. Copenhaver’s lab (University of North Carolina, USA) [29,30] were used. The FTL system is a powerful visual pollen assay based on segregation of genetically linked fluorescent proteins produced in pollen grains in a mutant background (*qrt^−/−^*) in which the pollen grains remain attached as tetrads, allowing the study of thousands of individual meiotic events directly, without analyzing the genome of the progeny. We selected Lines I5ab and I5cd both containing 3 genetically linked fluorescent markers on chromosome 5 [29,30]. These two lines have been extensively used [32,33,34,35,36,37,38,39,40] and allow recombination to be studied in the two intervals described in the previous section, which contain 21 and 1 NLR, respectively (Figure 1). The seeds of FTL lines I5ab and I5cd were a gift from Raphaël Mercier (INRAE Versailles, France).

### 2.3. Plant Growth Conditions

The FTL lines I5ab and I5cd used for the inoculation assay are hemizygous (presence/absence) for the three genetically linked fluorescent marker genes and homozygous for *qrt* mutation, in a homozygous Colombia background. The seeds were sowed in soil, vernalized for 2 days in the dark, at 4 °C and then grown in a greenhouse for 7 days (20 °C/12 h of light). Seven-day-old plantlets were then transferred to a growth chamber in long-day conditions (20 °C/16 h of light). Plants were maintained in high humidity conditions one-day before inoculation and during all the time of the experiment after infection.

### 2.4. Inoculation Assay with Pseudomonas syringae

**Figure 2:**
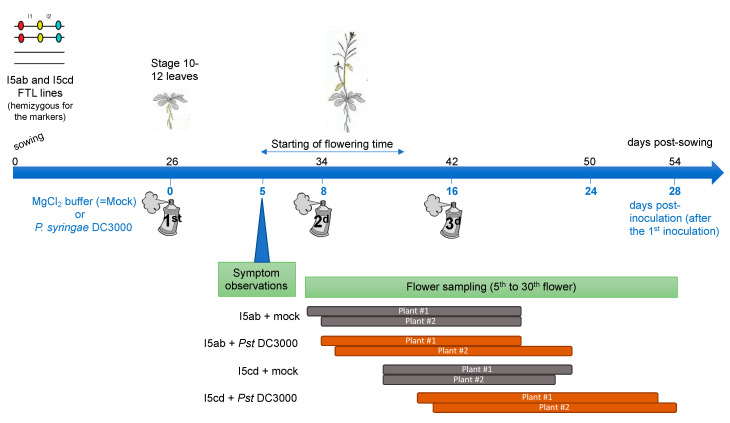
Temporal scheme of the experiment. At the stage 10-12 leaves, rosette leaves of FTL plants were sprayed either with mock-buffer or with *P. syringae* DC3000 bacteria. Symptoms were observed at 5 dpi. The 5th to the 30th flowers were collected on two plants for each FTL line and for each condition. In order to maintain pathogen pressure during all the experiments, two additional sprays with *P. syringae* DC3000 or with MgCl_2_ buffer were performed 8 (2^d^ inoculation, t = 8) and 16 days after the first inoculation spray (3^d^ inoculation, t = 16). For each inoculation, only the rosette leaves were inoculated, since the floral stem and the flowers were protected. The flowers were then collected at different time after an inoculation: at minimum 0 days after an inoculation, when sampling was done the same day than an inoculation, and at maximum 12 days, for the last collected flowers (at t = 28), since the 3^d^ inoculation was performed at t = 16.

At the stage 10–12 leaves, the plants were inoculated by the bacteria *Pseudomonas syringae* DC3000 (virulent) at O.D = 0.01 in MgCl_2_ 10 mM, by spraying the leaves, while protecting the center of the rosette (which will produce the future floral stem). As a control, plants were sprayed with MgCl_2_ 10 mM only. Nine and 18 plants were inoculated for each condition for line I5ab and for line I5cd, respectively. The same number of plants were used as a control, for each FTL line respectively. On each plant, symptoms were visually observed and the length of the floral stem was measured daily. To maintain a pathogen pressure during all the experiments, two additional sprays with *P. syringae* or with MgCl_2_ only were performed 8 and 16 days after the first inoculation spray (Figure 2). During the spray inoculation, the floral stem and the flowers were protected and consequently only the rosette leaves were inoculated.

### 2.5. Analysis of Pollen Tetrads of Fluorescent Tagged Lines

For two plants for each condition, the 5th to the 30th flowers were collected daily corresponding to a duration of 9 to 13 days of sampling according to the development of the plants (Figure 2). Days of sampling then corresponded to 0 to 12 days postinfection (after either the first, second or third inoculation) (Figure 2). The flowers were dropped in Pollen-sorting buffer (PSB), placed under vacuum during 20 min to facilitate imbibition of the buffer in the flowers and frozen until observation (adapted from [41].) Pollen tetrad slides were prepared as described in [30]. Automatic classification of the tetrads (A to L, see [30]) and counting were performed using the Metafer Slide Scanning Platform developed at INRAE Versailles [33] and then manually checked. Genetic distances (in cM) for each interval was calculated using the Perkins equation as described in Berchowitz et al. [30]. The Stahl Lab Online Tools (http://molbio.uoregon.edu/~fstahl/) was used for statistical analyses of the data.

## 3. Results

### 3.1. Selection of the Target Regions Based on the Available Arabidopsis FTL Lines

In order to investigate the potential impact of biotic stress on DNA recombination in plant meiotic cells, we selected the well-established pathosystem *Arabidopsis thaliana* and its interaction with the bacteria *Pseudomonas syringae* and took advantage of the existing *Arabidopsis* FTL. The selected FTL lines carry three genetically linked fluorescent markers of different colors (red, cyan and yellow), directed by a postmeiotic pollen-specific promoter. Moreover, FTL lines have been developed in a *qrt^−/−^* mutant background that produces pollen tetrads in which the four meiotic products are held together, allowing all products of a single meiotic event to be studied. In this *qrt^−/−^* homozygous background, by using hemizygous plants for the three genetically linked markers on the same chromosome, CO events can be directly detected in the pollen grains since in the tetrads, the fluorescent marker genes (and thus the proteins they encode) will segregate in patterns that reflect whether or not a recombination event has happened between them. In order to study genomic regions presenting a contrasting number of NLR genes, we selected two FTL lines referred to as I5ab and I5cd for inoculation assay. The I5ab interval (7.6 Mb) is located on the long arm of chromosome 5, and the I5cd interval (3.1 Mb) is located on the short arm of chromosome 5 (Figure 1). The two selected FTL lines contain both three fluorescent markers defining two adjacent intervals. The two adjacent intervals in the I5cd line contain no (I5d) or only one (I5c) NLR gene. In contrast, the target interval in the I5ab line contains a large number of NLR, with the I5a interval containing a cluster of 19 NLR genes and the I5b interval containing two isolated NLR genes.

### 3.2. Floral Stem Development is Slightly Delayed in Inoculated Plants

**Figure 3:**
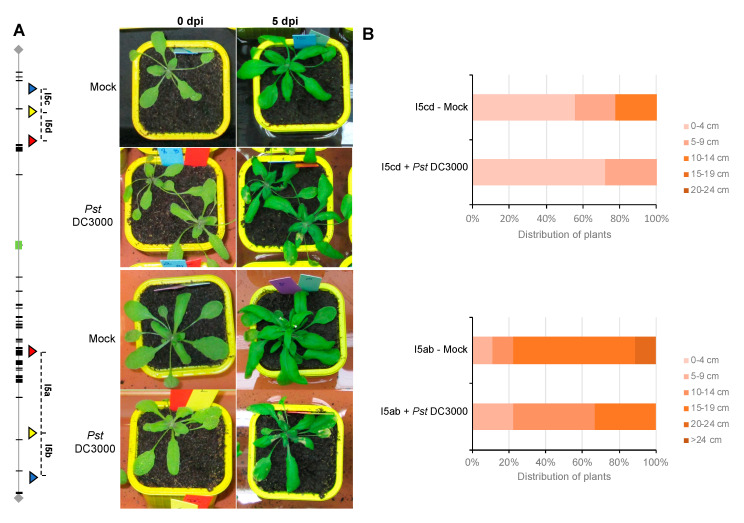
**Phenotype of FTL lines after infection with *P. syringae***. (**A**) Symptoms observed on *Arabidopsis* FTL lines after *P. syringae* infection at 5 dpi (after the first inoculation). The plants of FTL lines, I5cd (Upper panel) and I5ab (Lower panel), were sprayed with *P. syringae* DC3000 or with MgCl_2_ only as a control. Typical brown symptoms, including water-soaked lesions on the leaves, were visible at 5 dpi (after the first inoculation) on infected plants only and were still present when flowers were collected. (**B**) Floral stem development at 12 dpi (after the first inoculation) in mock- and in *Pst*-inoculated plants. The length of the floral stem was measured at 12 dpi on mock- or *Pst* DC3000- inoculated plants, for each FTL line, I5cd (Upper panel) and I5ab (Lower panel).

For both FTL lines, rosette leaves of three weeks-old plants were sprayed either with the virulent bacteria *Pseudomonas syringae* DC3000 or only with the buffer solution, as a control. Five days after the first inoculation (5 dpi), for both FTL lines (I5ab and I5cd), symptoms were visible on plants inoculated with *P. syringae*, whereas the control plants were completely healthy (Figure 3A). Both FTL lines (I5ab and I5cd) presented a shorter floral stem than mock plants 12 days postinoculation (Figure 3B and Figure S1). Thus, the growth and development of these lines were affected by pathogen attack.

### 3.3. Meiotic Recombination May Not Be Affected in Inoculated Plants

The impact of pathogen infection on meiotic DNA recombination was analyzed by microscopic examination of marker gene fluorescence in pollen tetrads from two mock and two inoculated plants of each FTL line (>30 flowers for each condition) (Table 1). For I5ab plants, around 500 pollen tetrads were scored for both mock and inoculated plants. For I5cd plants, we analyzed 600 pollen tetrads from infected plants and more than 700 tetrads from mock plants (Table 1). The tetrads were classified according to their fluorescence pattern and counted in each category (Table 1).

**Figure 4:**
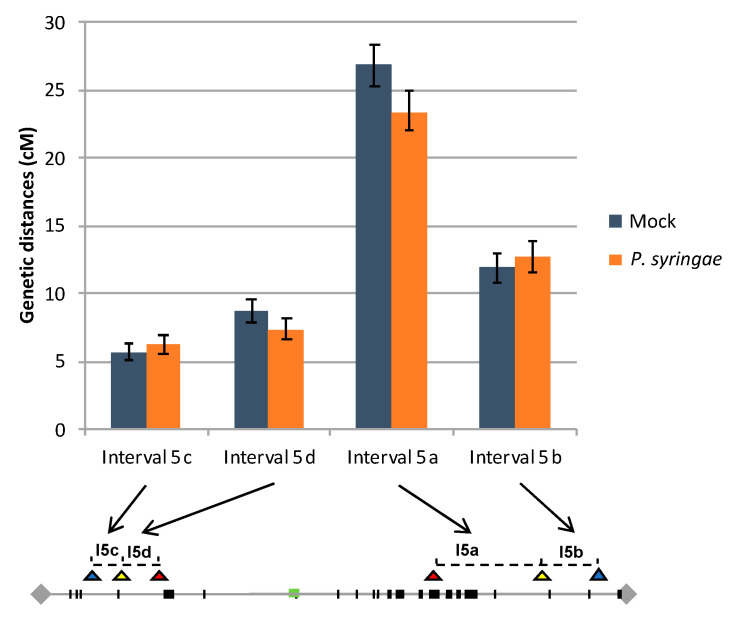
Recombination rate in control and infected plants. Recombination frequencies were calculated for each interval I5cd and I5ab, in infected and in control plants. The distribution of NLR genes and Fluorescent Tags on chromosome 5 are depicted in Figure 1 (see legends in Figure 1).

The recombination rates were calculated for each interval in both FTL lines using the standard Perkins genetic mapping equation and compared between control and infected plants [42]. Our data showed that in I5ab control plants, genetic distances were 26.9 +/− 1.6 cM and 11.9 +/− 1.1 cM for the interval I5a and I5b, respectively. Concerning the I5cd interval, in control plants, genetic distances were 5.7 +/− 0.7 cM for the interval I5c and 8.7 +/− 0.8 cM for I5d (Figure 4). All these values are in agreement with previously published values based on the same FTL lines in noninfected conditions [32,33,34,35,36,37,38,39,40]. In the *P. syringae* inoculated I5ab plants, the recombination rate observed for I5a (23.4 +/− 1.4 cM) and for I5b (12.8 +/− 1.2 cM) present no significant difference compared to the corresponding intervals in the I5ab control plants (Z test; *P* = 0.10 and *P* = 0.59 for I5a and I5b, respectively). Similarly, in the I5cd inoculated plants, the recombination rate observed in I5c (6.2 +/− 0.7 cM) and I5d (7.4 +/− 0.7 cM) intervals are not significantly different from the corresponding interval in the mock I5cd plants (Z test; *P* = 0.57 and *P* = 0.25 for I5c and I5d, respectively) (Figure 4). This suggests that in our experimental conditions, the biotic stress does not affect the meiotic process.

## 4. Discussion

In plants, it is now well-established that pathogen infection can increase somatic recombination not only at the site of infection but also in distant uninfected tissue thanks to an SRS produced at the local site of pathogen infection that can propagate systemically in the plant [21,25,43]. Consequently, it is conceivable that this SRS could reach the meiocyte cells and lead to an increased rate of meiotic recombination, favoring the generation of new *R* gene alleles. In the present paper, in order to test if pathogen infection has an impact on meiotic recombination, we took advantage of the *Arabidopsis* FTL lines. After infecting *Arabidopsis* leaves with the virulent bacteria *Pseudomonas syringae*, we found no clear evidence of a pathogen-associated meiotic recombination rate increase. Here we discuss potential explanations for this negative result that could be due to limitations related to the use of the FTL lines (analyses of specific intervals, on male meiosis only, the sensitivity of the experiments depending on the number of analyzed tetrads) or to the inoculation process or to the production of the SRS. We also discuss the implications of this finding, as well as alternative scenarios, that could be independent of the meiotic process.

First, despite its interest, both at the theoretical and applied levels, only a few studies have tried to tackle this difficult question by directly investigating the meiotic process in pathogen-infected plants [21,25,27,43]. In that context, *Arabidopsis* FTL lines constitute a powerful and cost-effective tool because it allows the analysis of thousands of individual meiotic events directly on pollen without generating a large progeny. However, one limitation of FTL lines is that it is not a genome-wide approach since it is focused only on selected intervals. Moreover, the fact that some genomic intervals may exhibit a recombination plasticity in response to some environmental cues but not others could be one possible explanation of our negative result. Indeed, this type of differential response depending on the intervals has been observed in *Drosophila* infected by the enterobacteria *Wolbachia* [44]. This hypothesis seems unlikely since our results were obtained within four different intervals located on chromosome 5 that define two large genomic regions of 7.6 Mbp (I5cd) and 3.1 Mbp (I5ab), presenting contrasting patterns in terms of NLR content. Nevertheless, we cannot completely rule out this possibility. Recent papers have proposed alternative genome-wide strategies to generate a precise map of COs such as high-throughput sequencing of progeny [45] or the sequencing of the pool of pollen DNA from an F1 plant [46]. These new strategies could be used in the future to address the impact of pathogen infection on meiotic recombination in a genome-wide manner. Compared to FTL, which is a cheap strategy once the required microscopy equipment is available, these two genome-wide strategies are more expensive and each has advantages and disadvantages. In particular, the high-throughput sequencing of progeny implies the tedious management of progeny, while the pollen strategy allows only to study male meiosis.

Second, we cannot exclude that bacterial infection performed in our experimental conditions may not impose sufficient stress to elicit a change in the frequency of meiotic recombination. Third, because of a statistical power issue, a very subtle increase in global COs rate after infection may have not been detected in our experimental conditions. However, a previous analysis performed with *Arabidopsis* FTL lines have shown that studying about 500–1000 tetrads allows the identification of a two- to three-fold increase in the recombination rate [37]. Consequently, since our analysis is based on a similar number of tetrads, a potential increase in the recombination rate as low as two- to three-fold should have been detected. If the recombination increase is lower than this range, this promotes the question of whether potential exploitation of this recombination increase might be possible in plant breeding. However, in an evolutionary context, even a modest increase in the recombination rate would be significant by increasing the probability of having a recombination event that would lead to a new NLR specificity.

Fourth, an inherent limitation of the *Arabidopsis* FTL lines, is that they are based on pollen tetrads analysis and consequently, they do not allow to address the possibility of a female-specific response to infection. A sex-related difference in COs distribution along chromosomes has been described in wild-type *Arabidopsis*. Indeed, COs distribution is heterogeneous, especially in subtelomeric regions where it exhibits very contrasting patterns between the male and female meiosis. However, globally, the five chromosomes undergo more COs in male meiosis than in female meiosis, suggesting that FTL analysis can be considered as a relevant tool in our study [47].

Fifth, so far, an increased somatic HR in noninfected tissues after biotic stress has been exclusively described in virus-infected plants. However, it is unlikely that the recombination response to infection could be pathogen-specific and may not be observed after bacterial infection like in the present study. Indeed, a systemic increase of somatic HR seems to be a general response observed not only after biotic stresses but also after various abiotic stresses. For example, in *Arabidopsis*, local γ irradiation on roots of young seedlings results in a significant and long-term increase of HR in the leaves in the aerial part of plants at the rosette stage, 20 days postirradiation [48]. Similarly, in Tobacco, plants treated with UVC, a well-known DNA damaging agent, or with Rose Bengal (RB) whose photoactivation leads to the production of reactive oxygen intermediates, exhibit a local and systemic increase of HR and grafting experiments demonstrated the existence of a systemic signal produced by UVC and RB exposure [49]. Consequently, these observations strongly suggest that in plants, the recombination signal propagation should correspond to a general response to both abiotic and biotic stresses and should not be specific to virus infection.

Finally, we cannot exclude that pathogen infection has no effect on meiotic recombination. This is in agreement with a previous work conducted in house mice infected by the pathogenic bacteria *Borellia burgdorferi*, causative agent of Lyme disease. Indeed, the analysis of the broad-scale patterns of COs distribution by MLH1 mapping revealed that the number of COs and the distribution of chiasmata are unaltered in infected male mice [50]. However, the link between the perception of stress and the generation of diversity has been exemplified in various organisms with a strong emphasis on arthropods, where several studies have shown that pathogen-infected females produced an increased proportion of recombinant offspring compared to the uninfected females. For example, in the fruit fly *Drosophila melanogaster,* a classical genetic approach based on molecular markers, revealed that infection by various bacteria (pathogenic *Serratia marcescens* and *Providencia rettgeri* or endosymbiotic *Wolbachia pipientis*) or by parasitic wasps significantly increased the recombinant progeny fraction [44,51]. Similarly, in mosquito *Aedes aegypti*, females infected by the microsporidian parasite, *Vavraia culicis*, were more likely to have recombinant offspring than the uninfected females [52]. Likewise, in plants, a higher frequency of plants presenting rearrangements was observed in the progeny of infected plants not only for a luciferase transgene [21] but also for *N*-homologs [27]. All these observations strongly suggest that during sexual reproduction increase of recombination helps to generate diversity in an organism’s offspring, to cope with an ever-changing array of pathogens.

In our study, the frequency of COs during meiosis in infected plants remains unchanged compared to control plants, while, as discussed above, several studies have described more recombinant progeny after pathogen infection. So how to explain this apparent discrepancy between our results centered on meiosis and previous studies centered on plant progeny analysis? First, we cannot rule out that the slower development of infected plants observed in our study could negatively impact the male recombination rate, counterbalancing a possible CO-promoting signal initiated by the pathogen stress. Moreover, several potential mechanisms may underpin the increased frequency of recombinant progeny, without involving an increase in the meiotic recombination rate. Indeed, the postinfection increase of recombinant offspring in plants could be caused by an increase of late mitotic recombination in germ cells, which would next undergo meiosis. In this latter case, this would have led to a bias in the frequency of certain recombinant tetrads that was not observed in our study. Alternatively, the infection-driven recombination response in plants could be mediated by transmission distortion of recombinant gametes rather than an overt increase in the meiotic recombination rate, as it has been proposed in the fruit fly, *Drosophila melanogaster* [51]. Although the molecular mechanisms of the distortion need to be elucidated, it could be due to differences in viability and/or in the meiotic behavior during segregation of the chromosomes of the recombinant gametes, compared to the nonrecombinant ones.

## 5. Conclusions

In this paper, using the powerful tool of FTL lines in *Arabidopsis thaliana*, we began to dissect the mechanisms underlying behind the stress-driven increase of recombinant offspring observed in plants [21,27,53], as predicted by the Red Queen model, which argues that sex is favored in the face of dynamic selection pressures. Clearly, investigations are still needed to understand the molecular mechanisms contributing to the recombination rate variation in the progeny of infected plants. Recently published genome-wide approaches such as high-throughput sequencing of progeny [45] or the sequencing of the pool of pollen DNA from an F1 plant [46] might be alternative efficient strategies to tackle this question in the future.

## Figures and Tables

**Table 1:** Classification and counting of pollen tetrads in control and inoculated plants of I5ab and I5cd lines. For each FTL line containing the three markers (cyan, red and blue), the different possibilities of markers distribution among the four chromatids and the corresponding distribution in the tetrad have been represented as drawings above each column, according to Berchowitz and Copenhaver’s nomenclature [30]. For each condition (control or inoculated), since a similar pattern of tetrad distribution was observed for the two plants studied, revealing no bias related to a single plant, the observed total number of each type of tetrad is given for the two plants corresponding, either to the interval I5ab (up) or to I5cd (down). Similarly, the total number of flowers and tetrads from the two control and the two inoculated plants analyzed in this study are also mentioned for both I5ab and I5cd lines.

**INTERVAL I5ab** 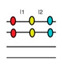			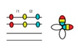	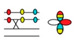	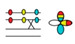	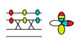	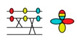	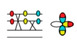	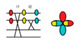	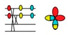	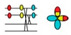	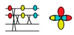	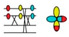	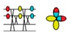	
Nb of tetrads	Nb of plants	Nb of flowers	0 CO	1 CO	1 CO	2CO	2CO	2CO	2CO	2CO	2 CO	3CO	3CO	4CO	
Tetrad Types			Class 1	Class 2	Class 3	Class 4	Class 5	Class 6	Class 7	Class 8	Class 9	Class 10	Class 11	Class 12	Total Tetrads
Control Plants	2	33	167	212	81	11	7	6	6	3	1	1	0	0	495
Inoculated plants	2	31	206	203	94	7	8	13	4	3	2	0	0	0	540
**INTERVAL I5cd** 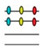			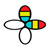	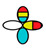	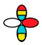	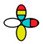	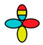	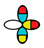	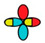	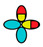	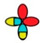	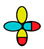	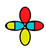	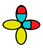	
Nb of tetrads	Nb of plants	Nb of flowers	0 CO	1 CO	1 CO	2CO	2CO	2CO	2CO	2CO	2 CO	3CO	3CO	4CO	
Tetrad Types			Class 1	Class 2	Class 3	Class 4	Class 5	Class 6	Class 7	Class 8	Class 9	Class 10	Class 11	Class 12	Total Tetrads
Control Plants	2	33	582	78	119	2	2	2	0	1	2	0	0	0	788
Inoculated plants	2	37	443	68	82	2	4	0	1	0	0	0	0	0	600

## Supplementary Materials

The following are available online at https://www.mdpi.com/2073-4425/11/7/832/s1, Figure S1: Symptoms observed on infected leaves of I5ab (upper panel) and I5cd (lower panel) plants at 5 dpi (after the first inoculation).
