# Supplementary material for: Deciphering the Impact of a Bacterial Infection on Meiotic Recombination in Arabidopsis with Fluorescence Tagged Lines
Source: Genes (Basel). 2020 Jul 21;11(7):832. doi: 10.3390/genes11070832 (PMC7397157; doi:10.3390/genes11070832)
Supplement: Supplementary file 1 [file genes-11-00832-s001.zip › genes-818980-supplementary-final.docx]

Supplementary Materials


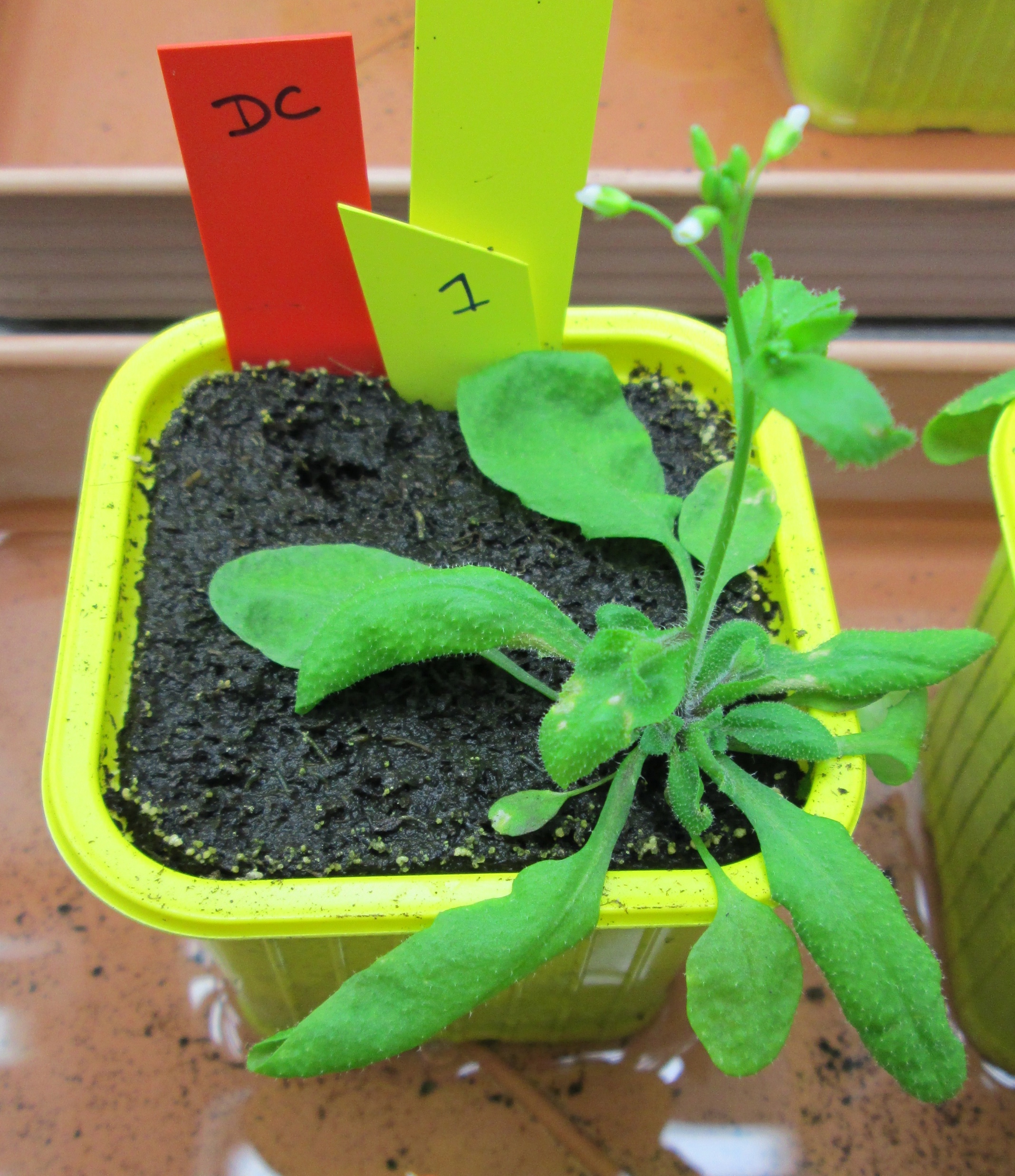

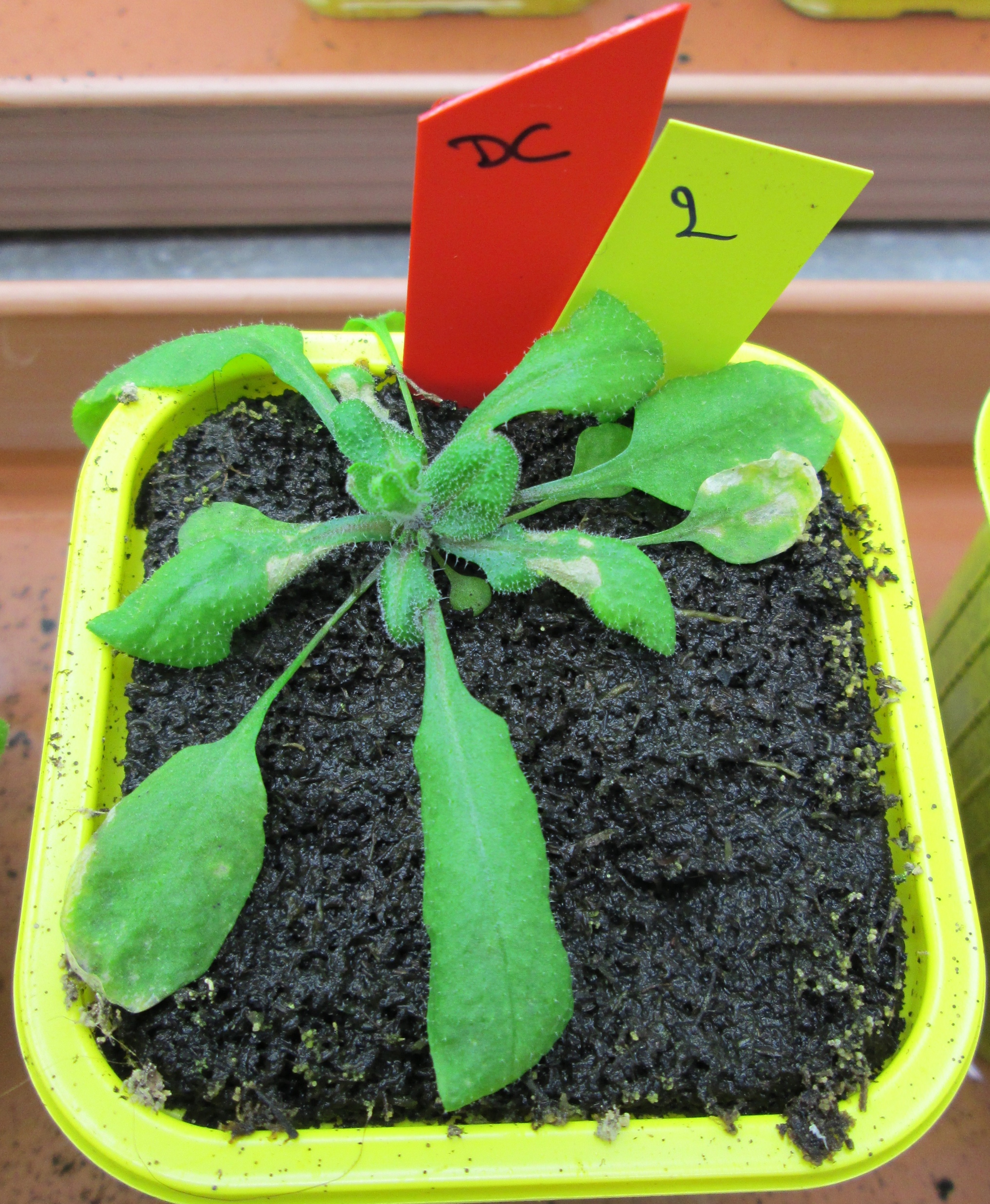

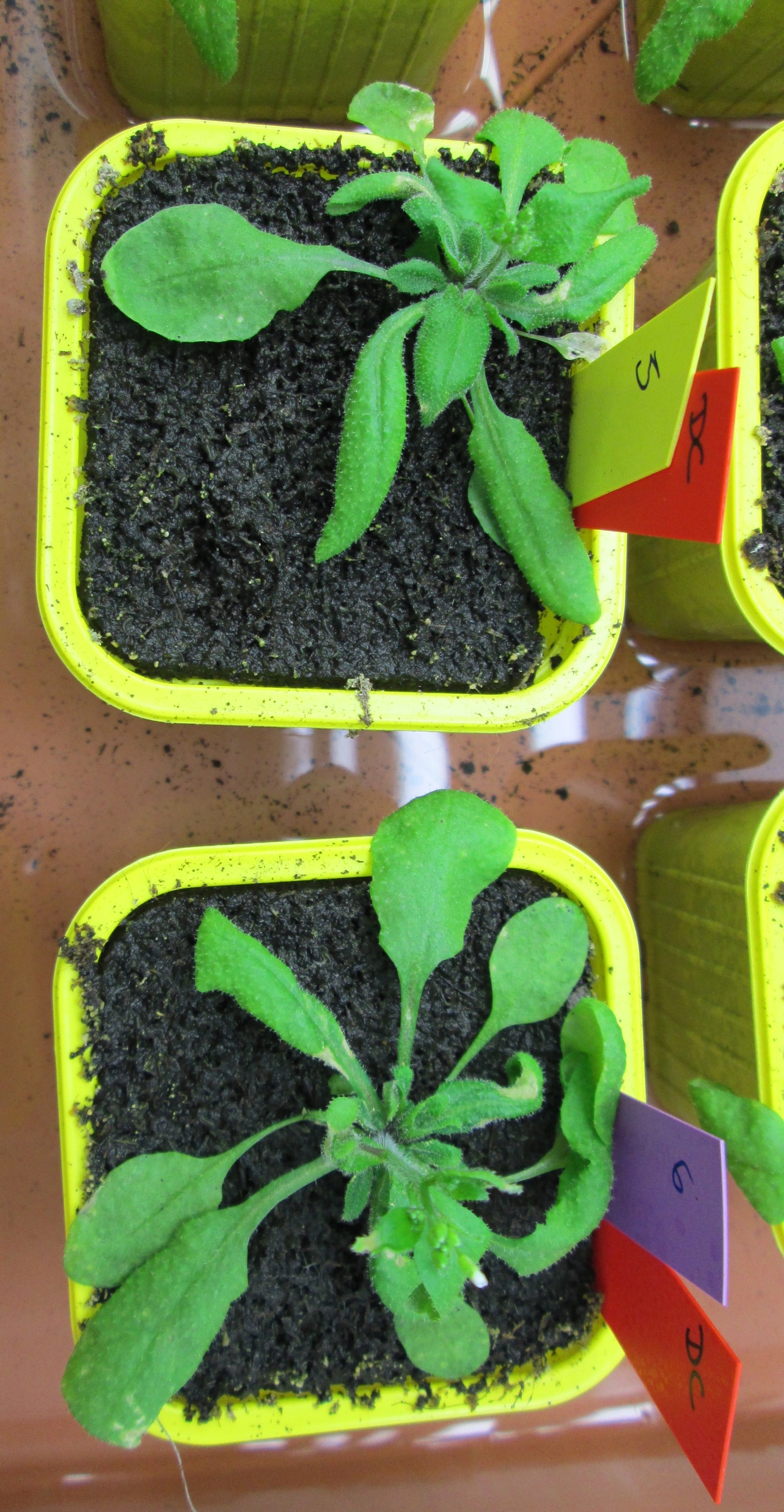

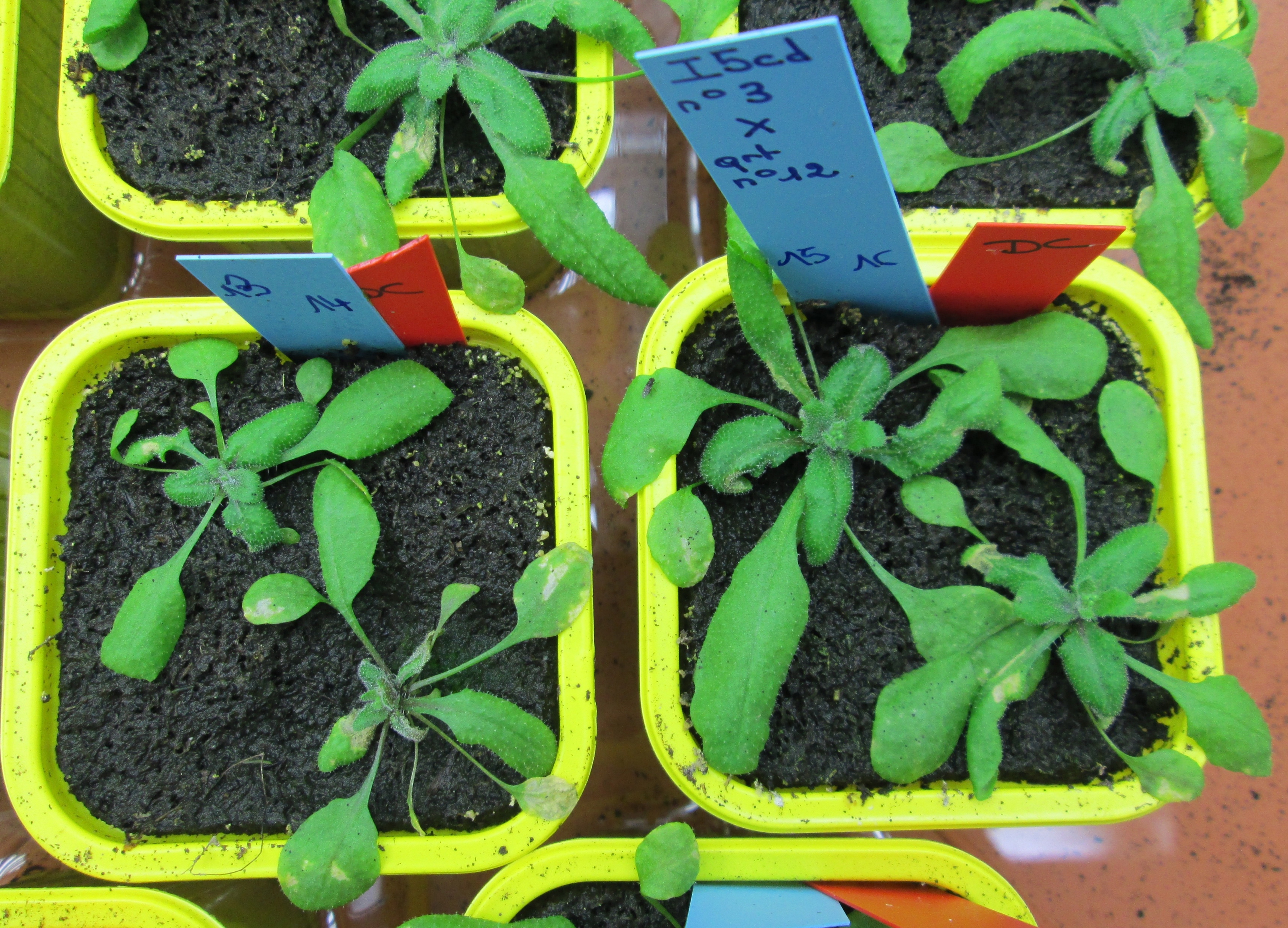

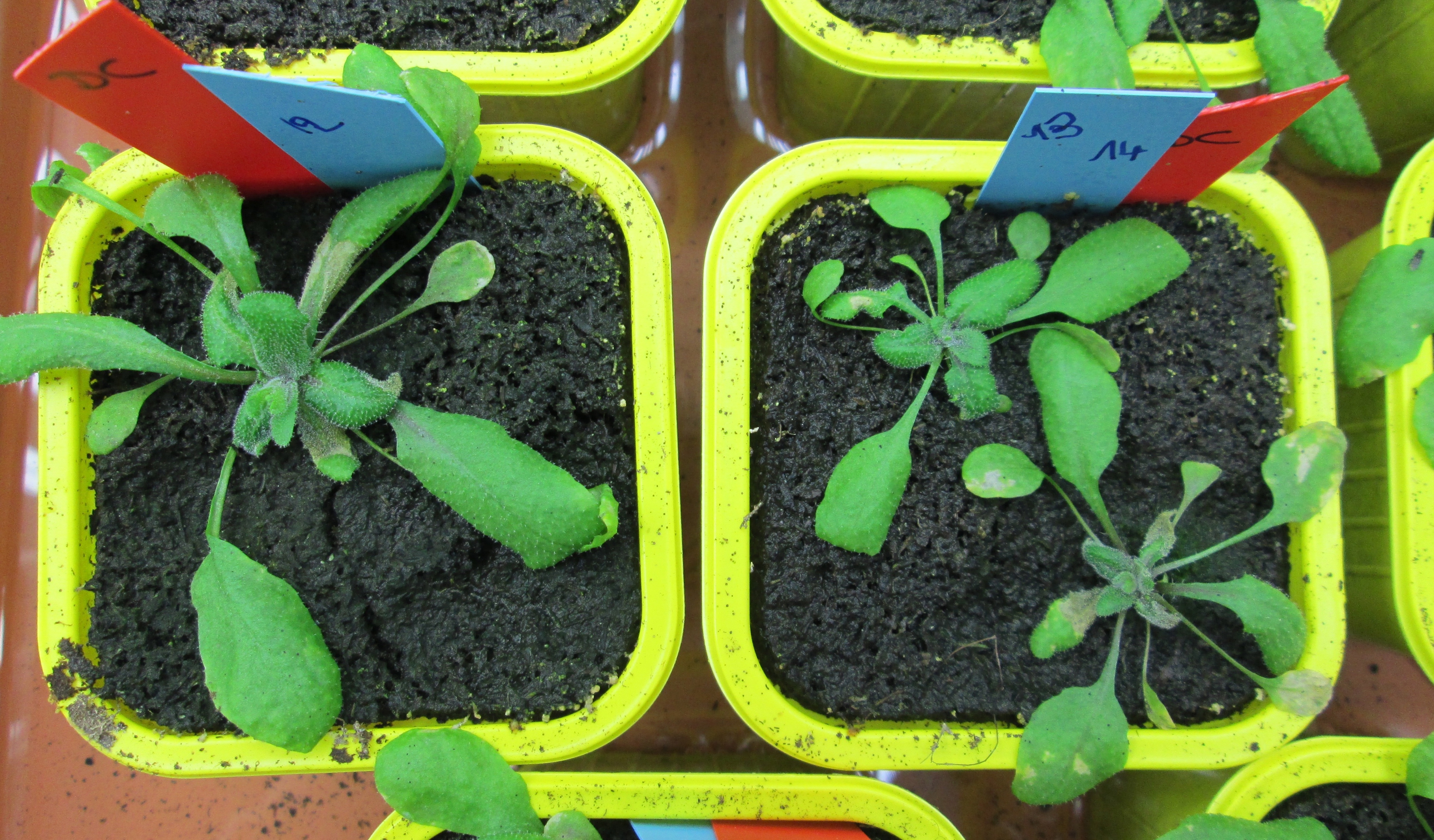


I5ab

I5cd

**Figure 1.** Symptoms observed on infected leaves of I5ab (upper panel) and I5cd (lower planel) plants at 5 dpi (after the first inoculation). The rosette leaves of I5ab and I5cd plants were spray-inoculated with *Pst* DC3000 (or with mock buffer as control, not presented on this figure, see Figure 2), taking care to protect from the spray the center of the plants (futur floral stem). Five-days after, symptoms were observed only on the infected plants, presenting typical water-soaked lesions. No symptoms were observed on control plants (not presented).
